# Vascular Cognitive Impairment through the Looking Glass of Transcranial Magnetic Stimulation

**DOI:** 10.1155/2017/1421326

**Published:** 2017-02-26

**Authors:** Giuseppe Lanza, Placido Bramanti, Mariagiovanna Cantone, Manuela Pennisi, Giovanni Pennisi, Rita Bella

**Affiliations:** ^1^Department of Neurology IC, I.R.C.C.S. “Oasi” Institute for Research on Mental Retardation and Brain Aging, 73 Via Conte Ruggero, 94018 Troina, Italy; ^2^I.R.C.C.S. Centro Neurolesi “Bonino-Pulejo”, Via Provinciale Palermo, Contrada Casazza, 98124 Messina, Italy; ^3^Spinal Unit, Emergency Hospital “Cannizzaro”, 829 Via Messina, 95126 Catania, Italy; ^4^Department of Surgery and Medical-Surgical Specialties, University of Catania, 78 Via S. Sofia, 95123 Catania, Italy; ^5^Department of Medical and Surgical Sciences and Advanced Technology, Section of Neurosciences, University of Catania, 78 Via S. Sofia, 95123 Catania, Italy

## Abstract

In the last years, there has been a significant growth in the literature exploiting transcranial magnetic stimulation (TMS) with the aim at gaining further insights into the electrophysiological and neurochemical basis underlying vascular cognitive impairment (VCI). Overall, TMS points at enhanced brain cortical excitability and synaptic plasticity in VCI, especially in patients with overt dementia, and neurophysiological changes seem to correlate with disease process and progress. These findings have been interpreted as part of a glutamate-mediated compensatory effect in response to vascular lesions. Although a single TMS parameter owns low specificity, a panel of measures can support the VCI diagnosis, predict progression, and possibly identify early markers of “brain at risk” for future dementia, thus making VCI a potentially preventable cause of both vascular and degenerative dementia in late life. Moreover, TMS can be also exploited to select and evaluate the responders to specific drugs, as well as to become an innovative rehabilitative tool in the attempt to restore impaired neural plasticity. The present review provides a perspective of the different TMS techniques by further understanding the cortical electrophysiology and the role of distinctive neurotransmission pathways and networks involved in the pathogenesis and pathophysiology of VCI and its subtypes.

## 1. Background

The modern concept of vascular cognitive impairment (VCI), which encompasses any degree of vascular-related cognitive decline [1], is deemed to be the most common cognitive disorder, with a growing impact on social and health care expenses [2]. Moreover, early onset of VCI is also highly frequent in older stroke survivors, as showed in different studies [3–5]. The VCI construct includes not only vascular dementia (VaD), but also mixed dementia (vascular and degenerative) and vascular cognitive impairment-no dementia (VCI-ND), which refers to a subgroup of patients who manifest cognitive decline resulting from cerebrovascular injury but do not satisfy the diagnostic criteria of dementia [1, 2]. In addition to cognitive impairment, mainly involving processing speed and executive functioning [6], VCI patients also show behavioral (i.e., apathy, irritability, psychomotor agitation, disinhibition, and aberrant motor behavior) and mood deficits (namely depression, with or without anxiety) that correlate with worsening of both cognitive and functional status [7]. Moreover, strokes of the basal ganglia and internal capsule increase significantly the risk of poststroke depression and executive dysfunction [8]. Dementia after stroke may encompass all types of cognitive disorders [9], whereas a state of cognitive dysfunction before the index stroke is termed “pre-stroke dementia,” which may entail vascular changes as well as insidious neurodegenerative processes.

As known, white matter hyperintensities, commonly seen on brain T2-weighted magnetic resonance imaging (MRI), are associated with varying degrees of cognitive impairment in patients with stroke, cerebral small vessel disease, and dementia [10], although the pathophysiological mechanisms within the white matter accounting for cognitive dysfunction remain unclear. Nevertheless, the strong relationship between vascular white matter lesions (WMLs) and nonmotor sequelae has been established in large community-based populations [11–14], showing that cognitive and mood-behavior abnormalities may arise from the ischemic disruption of the prefrontal cortical-subcortical circuits [15]. Medial temporal lobe atrophy was also found to be a significant imaging predictor of early cognitive dysfunction in stroke survivors [16]. A novel association between irreversible astrocyte injury and disruption of gliovascular interactions at the blood-brain barrier in the frontal white matter and cognitive impairment in elderly poststroke survivors has been recently proposed. In particular, clasmatodendrosis was suggested as another pathological substrate linked to frontal white matter hyperintensities, which may contribute to poststroke or dementia due to small vessel disease [17].

However, cognitive dysfunction and functional limitations are also associated with depressive disorder in stroke survivors [3, 5, 7, 18]. Even subcortical ischemic vascular disease, including silent lacunar infarcts and WMLs, may be associated with late-life depression, often referred as “vascular depression” [19]. In 1997, Alexopoulos et al. [20] named “depression-executive dysfunction syndrome of late life” a clinical picture characterized by psychomotor retardation, difficulties at work, apathy, lack of interest, and limited depressive ideation and insight, together with prominent executive dysfunction at neuropsychological tests (i.e., planning, working memory, and set-shifting).

Brain imaging widely support these findings and provide the neuroradiological correlate of VCI and vascular depression [21–23]. Patients with vascular depression associated to WMLs show distinctive clinical-psychopathological findings with respect to those with lacunar lesions [13, 19, 24–28], with different prognostic implications as well [21, 26, 29]. In particular, whereas depressive symptoms are similar between the two groups, executive dysfunction and deficit of information processing speed are more frequently reported in patients with WMLs than in those with lacunar state [19, 25, 26]. Moreover, depressed subjects with WMLs showed a more rapid decline of cognitive and motor performances, as well as the presence of gait abnormalities and urinary disturbances [24]. It is also noteworthy that the severity of subcortical WMLs, rather than lacunar state, is associated with development of depressive symptoms [28] and to a history of late-onset depression [13]; similarly, microstructural white matter abnormalities of frontostriatal-limbic networks are related to executive dysfunction and late-life depression [25]. Finally, different reports emphasize that WMLs and executive dysfunction are linked to both poor response to treatment and progression to chronic depression compared to those with lacunar infarcts [21, 26, 29].

Although the understanding of different aspects concerning VCI grows over time, as reflected into a considerable body of literature published every year, VCI still remains a broad concept whose borders are not always well defined [30]. Probably, the most pressing challenge is the difficulty in discriminating the different forms of VCI and in recognizing the earliest stages from normal brain aging [31, 32]. Currently, the diagnosis of VCI, whether at the stage of VCI-ND or an overt picture of VaD, is mostly based upon clinical and neuropsychological evaluation, together with structural and functional neuroimaging. The pathological diagnosis of suspected clinical VaD requires adequate postmortem brain sampling and rigorous assessment methods to identify the commonly observed substrates [33]. The need for screening and early diagnosis tools have focused the search to identify early biological and instrumental markers of disease process and progress. In this context, systematic neurophysiological assessment of VCI can aid the diagnosis and predict the response to drugs. Among neurophysiological techniques, transcranial magnetic stimulation (TMS) emerged as valuable noninvasive method for the functional evaluation of cerebral cortex and neurotransmission pathways involved in a number of psychiatric and neurological disorders, including cognitive impairment and dementia [34].

The present review provides a perspective of the different TMS techniques by further understanding the cortical electrophysiology and the role of distinctive neurotransmission pathways and networks involved in the pathogenesis and pathophysiology of VCI and its subtypes.

## 2. Transcranial Magnetic Stimulation: Basic Principles and Application in Cognitive Neuroscience

Clinically introduced approximately 30 years ago as a diagnostic tool to study the central motor pathways, today, TMS goes well beyond the simple assessment of the corticospinal tract. Indeed, it is able to provide novel insights into the pathophysiology of the neural circuitry underlying neurological and psychiatric diseases and to give in vivo information about the excitability of the human brain cortex and the conduction along corticospinal tract as well as the functional integrity of intracortical neuronal and callosal fibers [35–37]. TMS has also a strong talent to unveil and monitor motor system impairment in the preclinical phase of several neurological disorders [34] or systemic diseases with the central nervous system (CNS) involvement [38, 39]. Moreover, integrated approaches using neurophysiological techniques together with structural and functional imaging have allowed us to study the connectivity across motor and nonmotor areas [40–43]. Finally, by evaluating the effects of agonists or antagonists for specific neurotransmitters, TMS can selectively and noninvasively explore the function of glutamatergic, gamma-aminobutyric acid- (GABA-) ergic, monoaminergic, and cholinergic central circuits (the so-called “pharmaco-TMS”) [44, 45].

TMS can be delivered to the same or different brain areas as single pulse, pairs of stimuli, paired cortical and peripheral stimulation, or as trains of repetitive stimuli at various frequencies.

### 2.1. Single-Pulse TMS

A single magnetic pulse applied to the primary motor cortex at adequate stimulator intensity elicits a motor evoked potential in contralateral target muscles, thus providing a functional assessment of the corticospinal conduction [46]. In particular, the latency of motor evoked potentials and the central motor conduction time, defined as the time interval difference between the motor evoked potentials induced by motor cortex stimulation and those evoked by motor root stimulation, are indexes of integrity of the corticospinal pathways. The motor evoked potential amplitude reflects an aggregate measure of the excitation state of output cells in the motor cortex, nerve roots, and the conduction along the peripheral motor pathway to the muscles [47].

The resting motor threshold is considered a global parameter of human brain excitability, as it is a compound measure of the membrane excitability of cortical motor neurons, neural inputs into pyramidal cells within the cortex, as well as the excitability of spinal motor neurons, neuromuscular junctions, and muscles [47, 48].

A suprathreshold TMS pulse applied to the primary motor cortex during a tonic voluntary contraction of contralateral muscles results in a suppression of the electromyographic (EMG) activity of those muscles lasting few hundred milliseconds [49]. This phenomenon, called contralateral cortical silent period, is exploited as a functional measure of intracortical inhibitory circuits [50, 51], mainly mediated by GABA-B transmission [52]. Conversely, the ipsilateral silent period, evoked by stimulating the muscle and hemisphere of the same side, is considered to reflect the interhemispheric corticocortical inhibitory mechanisms, and it is thought to be modulated by transcallosal output neurons projecting to contralateral GABA-ergic interneurons [35, 36].

### 2.2. Paired-Pulse TMS

The paired-pulse TMS paradigm [53] allows the assessment of measures of intracortical interneuronal functions and interactions, such as the short-latency intracortical inhibition and intracortical facilitation of the motor response [53, 54]. Short-latency intracortical inhibition is probably mediated by the activity of intracortical GABA-A interneurons [55]; intracortical facilitation is more complex being probably related to the activation of a cortical circuit projecting upon corticospinal cells different from that preferentially activated by single-pulse TMS, and it seems dependent to a great extent on the activity of glutamatergic excitatory interneurons [56, 57].

### 2.3. Sensory-Motor Modulation and Plasticity

Using specific TMS techniques, it is possible to investigate the sensory-motor interaction within the cerebral cortex as well as the cortical phenomenon of the short-latency afferent inhibition and long-latency afferent inhibition. Short-latency afferent inhibition is thought to reflect the integrity of central cholinergic neural circuits, as it has been shown to be reduced or abolished by the muscarinic antagonist scopolamine in healthy subjects [58, 59] whereas it is positively modulated by acetylcholine [60]. It has been suggested that it may depend on the integrity of circuits linking sensory input and motor output [61], given that other neurotransmitters (such as dopamine) are supposed to play a modulatory role in the cholinergic transmission [62]. Long-latency afferent inhibition is probably related to the cortical-cortical connections involving the motor cortex and both primary and secondary somatosensory cortical areas [61].

TMS also allows us to study the synaptic plasticity phenomena at different levels. In healthy subjects, a magnetic stimulus applied after a brief period of exercise reveals phenomena of “post-exercise facilitation” and “delayed facilitation” that provide valuable information on cortical excitability and intracortical synaptic reorganization underlying motor learning [63].

### 2.4. Repetitive TMS

Single TMS pulses delivered in trains are the principle of repetitive TMS (rTMS), an approach that can transiently modulate the functioning of stimulated and connected brain areas mainly depending on the frequency of stimulation [36, 64, 65]. For this reason, rTMS might have therapeutic and rehabilitative applications since the effects of repeated sessions may persist in time [40, 48, 66–70]. The mechanisms of these changes are not completely clear but seem to be related to the phenomena of synaptic long-term potentiation (LTP) and long-term depression (LTD) within the CNS [71, 72]. Similarly, it is possible to induce LTP-like changes in the sensory-motor system by means of the paired associative stimulation [73], which induces a lasting increase of corticospinal excitability that can be considered as a marker of cortical plasticity [73, 74].

### 2.5. TMS in Dementia

Although the single neurophysiological abnormality revealed by TMS is not disease-specific [36], distinctive set of electrocortical changes that cosegregate specifically in different neurological and psychiatric disorders has been found, suggesting the involvement of specific biological substrates in their pathogenesis [34, 75, 76]. In the last years, a number of studies have shown abnormalities in TMS assays of cortical function in dementias, namely an increased excitability of the motor cortex in both Alzheimer's disease (AD) [77] and VaD [78].

Even if not always clinically evident, the involvement of motor areas in dementia has been demonstrated by clinical, neuropathological, and neuroimaging studies. Changes in motor areas may be secondary to the direct structural alterations caused by the disease process but, more often, they are the consequence of indirect remodeling mechanisms [34]. In this context, increasing evidences support the hypothesis that the phenomena of brain plasticity are involved in different kinds of dementia, related to functional and structural components, each entailing a number of cellular mechanisms operating at different time scales, synaptic loci, and developmental phases within an extremely complex framework [79]. However, the exact relationship between brain plasticity and excitability of cortical areas and their connections is not completely understood yet.

Table 1 summarizes all the TMS studies in patients with VCI and its subtypes [76, 80–97].

## 3. TMS Correlates of VCI Subtypes

### 3.1. TMS in Vascular Dementia

The majority of TMS studies indicate that the motor cortex of VaD patients is hyperexcitable (reduced resting motor threshold) [78], a common feature shared by AD [77]. This finding has been considered as part of a plastic compensatory mechanism in response to neuronal loss and/or ischemic injury [34, 40]. Accordingly, the enhanced excitability and plasticity might counteract cognitive decline and shed light on the reasons underlying decline or preservation of cognitive domains in dementing population [87, 88]. This hypothesis has been demonstrated by means of TMS mapping study in AD patients, which showed a frontal and medial shift of the motor cortical output center of gravity, suggesting a functional reorganization of cortical brain areas, at least in the early stages [98]. A similar pattern has been also observed in subcortical ischemic VaD [84], which encompasses a homogeneous subtype of patients of particular interest because of the relatively slow progression, often making the differentiation from AD difficult [1, 2].

However, although a cortical reorganization similar to that occurring in AD was hypothesized also in VCI, it was not clearly demonstrated yet. Based on this assumption, Guerra and coworkers explored the relationship between excitability and plasticity in subcortical ischemic VaD [94]. Although obtained from a small sample size, they found that motor cortex had enhanced excitability in patients with AD and subcortical ischemic VaD with respect to controls. However, more interestingly, the motor cortex was plastically rearranged in both groups of patients, although with a slightly lesser center of gravity frontal shift in those with subcortical ischemic VaD compared to AD. Moreover, a significant direct correlation between parameters associated to cortical excitability and those associated to cortical plasticity was evident [94]. This would suggest the existence of mechanisms that partially overlap and probably act in the same neurophysiological way although they are, at least in principle, different both in location (subcortical versus cortical) and origin (vascular versus degenerative). The authors concluded that AD and subcortical ischemic VaD can share a common neurophysiological platform, related to the progressive neuronal loss within motor areas and to the ischemic disconnection, respectively [94]. This alteration could finally promote a functional rearrangement that allows the preservation of motor programming and execution despite disease progression [90, 94].

### 3.2. TMS in Vascular Cognitive Impairment-No Dementia

A crucial issue is whether it is possible to early identify VCI-ND subjects at risk for clinical progression. In a previous study on nondemented elderly patients with subcortical vascular disease and clinical-cognitive profile of VCI-ND [87], it was found that the ischemic interruption of prefrontal-subcortical loops implicated in executive functions and mood/affection regulation might result in functional changes of intracortical excitatory neuronal circuits, specifically an enhanced intracortical facilitation. Moreover, it has been shown that transcallosal inhibitory mechanisms were spared in patients with leukoaraiosis, a finding which differs from those with degenerative dementia (such as AD), in whom a functional callosal impairment is observed even in the preclinical or early stages [89].

A pilot TMS study after a 2-year follow-up has been performed on the same participants with VCI-ND [88]; compared to baseline, they showed an increase of global cortical excitability (reduction of the median resting motor threshold), along with a significant worsening of neuropsychological tests evaluating frontal lobe abilities, although without reaching a substantial functional impairment. We considered these findings to be part of the plastic compensatory mechanisms following the loss of motor cortical neurons, supporting the concept of VCI as a dynamic condition and some TMS changes as indexes of motor cortex plasticity [74]. Accordingly, it has been hypothesized that the critical point at which the resting motor threshold becomes abnormal might represent a “neurophysiological cut-off” to discriminate VCI patients advancing to VaD from those cognitively stable. If these patients show a “hyperfacilitation” at baseline, this would be considered as a trend of electrocortical dysfunction in “brain at risk” during the transition from normal brain aging to VCI, up to an overt VaD [30, 88].

More recently, intriguing findings come from the investigation of central cholinergic circuit functioning by means of short-latency afferent inhibition. Unlike AD, the role of cholinergic involvement in VaD is indeed still under debate, and TMS data are limited and often conflicting [81, 82, 85]. In a recent paper aiming at the evaluation of short-latency afferent inhibition in a sample of VCI-ND patients with predominant WMLs, central cholinergic circuitry was found to be not clearly involved in patients compared to age-matched controls [95], suggesting a distinctive profile of the cholinergic pathway with respect to primary cholinergic forms of dementia (namely, AD), even in the early stage [99]. A reasonable explanation is that VCI may exhibit considerable interindividual variation in the location of subcortical infarcts and, therefore, in the distribution and magnitude of the resultant cholinergic denervation [100]. This study also supports the role of short-latency afferent inhibition in providing useful insight in the diagnosis and prognosis of different dementing process and in the identification of responders to acetylcholinesterase inhibitors [95].

### 3.3. TMS in Vascular Depression

Also at the TMS level, available data support the “vascular depression hypothesis” as a different syndrome with respect to nonvascular early-onset major depressive disorder. In particular, the depressive syndrome in vascular depression should be regarded as one of the clinical manifestations in the wide symptom spectrum of subcortical cerebrovascular disease rather than being a primary disease [76, 86]. Consistent with this hypothesis, an increased intracortical facilitation was observed not only in nondepressed VCI patients but also in those with late-onset vascular depression [76, 86]; conversely, the level of intracortical facilitation in patients with early-onset major depression and without evidence of cerebrovascular disease was similar to healthy controls [76]. This result suggests that the disruption of frontal-striatal circuits by vascular lesions may predispose, precipitate, or perpetuate late-life depression. In particular, the vascular “disconnection” at the level of the dorsolateral prefrontal cortex (DLPFC) or the dorsal portion of the head of the caudate nucleus may affect the presentation and course of vascular depression [29].

However, at present, little is known about the impact of late-onset depression on plastic changes that could contribute to the preservation of cognitive functions in VCI patients. To better characterize the possible role of depression in cognitive decline of patients with vascular damage, we have recently investigated the relationship between the progression of the neurophysiological changes and cognitive impairment in patients with VCI-ND with those obtained in a group of patients with vascular depression and controls [96]. A high level of intracortical facilitation at baseline was found in nondepressed patients only, and it has been considered to be protective from cognitive decline, possibly through an enhancement of glutamate-related neuroplasticity. At follow-up, a hyperexcitability was observed in both groups of patients, pointing out an involvement of glutamatergic neurotransmission as well, but without a specific neurophysiological change that parallels cognitive decline in depressed patients. This indicates that the mechanisms that contribute to cognitive deterioration in vascular depression might be related either to subcortical changes produced by vascular lesions or to the lack of compensatory functional cortical [96].

### 3.4. TMS in CADASIL

The cerebral autosomal dominant arteriopathy with subcortical infarcts and leukoencephalopathy (CADASIL) is due to mutations in the *Notch3* gene on chromosome 19 and causes progressive cognitive decline till dementia, cerebral ischemic events, psychiatric disorders, and migraine. It reasonably represents a pure genetic model of VaD; these patients are therefore particularly suitable for exploring the relationship between ischemic brain injury, clinical manifestations, and TMS changes [78].

In 2008, Manganelli et al. [83] first demonstrated a dysfunction of motor cortex excitability and cholinergic innervations in terms of reduced resting motor threshold and short-latency afferent inhibition. The recurrent vascular insults and the lesion burden in strategic areas typically involved in CADASIL [101], such as the external capsule [102], may lead to the interruption of corticosubcortical cholinergic circuits. Therefore, it has been hypothesized that the cortical hyperexcitability in CADASIL might be caused by cholinergic, GABA-ergic, and glutamatergic neuron dysfunction, supporting the hypothesis of several neurotransmitter system involvement in CADASIL, similar to other forms of dementia [78]. More recently, short-latency afferent inhibition was found to be significantly reduced in both AD and CADASIL patients, although administration of L-3,4-dihydroxyphenylalanine (L-Dopa) was able to significantly increase short-latency afferent inhibition in the AD group only, suggesting that relationship between acetylcholine and dopamine systems may be specifically abnormal in AD [92].

The study of different intracortical circuits and sensory-motor plasticity using TMS protocols confirmed that acetylcholine and glutamate are involved in CADASIL and that abnormal plasticity correlates with the neuropsychological profile [91]. Similarly, cathodal transcranial direct current stimulation (tDCS) revealed no evidence of cortical dysplasticity in CADASIL, suggesting that increased rapid-onset cortical plasticity may contribute to largely preserve cognitive and motor functions despite extensive ischemic subcortical vascular disease [103].

## 4. The Contribution of Neuromodulatory Techniques

Repetitive TMS and tDCS are emerging as promising tools to modulate cortical circuits and related neurochemical pathways in dementing illnesses [104–106]. Several studies, although methodologically heterogeneous, have shown that specific paradigms of stimulation might improve cognitive performance and mood-behavioral symptoms, possibly becoming an alternative to conventional neuroleptic therapy for psychiatric symptoms of dementia [104]. Current pharmacological treatment, indeed, suffers from significant limitations, such as nonspecific mode of actions, an insufficient tailoring to the individual, and a number of adverse effects. The targets for an ideal nonpharmacological neuromodulatory treatment would be (a) modulation of activity in the targeted area, (b) modulation of activity in a dysfunctional network, (c) restoration of adaptive balance in a disrupted network, (d) guiding plasticity for best behavioral outcome, and (e) suppression of maladaptive changes for functional advantage.

In AD, there is a general trend for improvements across a wide range of cognitive outcome measures following treatment with rTMS and tDCS, probably mediated by compensatory mechanisms supporting the residual abilities, even with long-lasting effects [104]. Typical sites of stimulation include the DLPFC, temporal regions, temporoparietal regions, or a combination of multiple regions. Interestingly, the benefits may be highly task-specific (i.e., action naming versus object naming and visual recognition versus spatial recognition), taking into account that dementia severity may affect the clinical response. Combining these techniques with cognitive rehabilitation might influence learning in a neuroplastic fashion [104].

In patients with vascular-related cognitive decline, rTMS data are very few. In a randomized controlled pilot study on patients with subcortical ischemic vascular disease and clinical picture of VCI-ND, Rektorova et al. [107] showed that high-frequency stimulation over the left DLPFC improved executive performance and hypothesized that long-lasting effects could be due to an indirect activation of monoaminergic neurons located in the midbrain (dopamine) and/or the brainstem (noradrenaline and serotonin) and their cortical and subcortical targets [107]. Among individuals with vascular depression, rTMS is mentioned as a nonpharmacological option, although WMLs load and global vascular risk profile are predictors of poor response [108]. Finally, a recent systematic review evaluating the effectiveness of rTMS in improving vascular depression and poststroke depression concluded that it was beneficial in treating depression among individuals with cerebrovascular disease over the short term. However, heterogeneous populations and variability in study design and protocol as well as a limited number of investigations challenge the ability to draw firm conclusions on the effectiveness of rTMS [109].

Very recently, exciting results come from preclinical studies showing the restorative effect of rTMS on cognitive ability in murine model of VaD and its impacts on hippocampal synaptic plasticity [110, 111]. In this context, another possible mechanism of action of noninvasive brain stimulation in dementia is represented by the modulation of neurotrophin release. Indeed, experimental studies in rat models of AD have shown that tDCS can improve learning in mice through secretion of brain-derived neurotrophic factor (BDNF) and activation of tyrosine kinase B receptor [112]. Moreover, low-frequency rTMS might improve cognitive deficits through the upregulation of the hippocampal BDNF and the expression of the glutamate receptor for N-methyl-D-aspartate (NMDA) [113]. Finally, low-frequency rTMS in VaD model rats may improve learning and memory, protect pyramidal cells from apoptosis, and promote hippocampal synaptic plasticity through increased expression of the *Bcl-2* and reduced expression of *Bax* [114].

## 5. Limitations, Critical Aspects, and Possible Solutions

Although innovative, the approach based on noninvasive brain stimulation in vascular-associated cognitive impairment includes a number of potential criticisms:

- One is difficulties in recruitment of a sufficient number of age-matched controls without evidence of cerebrovascular disease at neuroimaging (that is strikingly prevalent among elderly) or with any cognitive impairment at neuropsychological evaluation.
- The correlation between different TMS patterns of cortical excitability and anatomical distribution and severity of vascular lesions has not been systematically investigated; therefore, without the contribution of advanced imaging, neuronavigated systems, or other electrophysiological techniques (i.e., high-density EEG), the spatial resolution of TMS remains quite low. However, the majority of patients enrolled in the studies here reviewed exhibited predominant WMLs within the frontal lobes, thus partially limiting this variability.
- TMS-related measures of cortical excitability do not provide specific clinical information but are sensitive to the “global weight” of several neurotransmitters, as well as to subcortical and cortical motor inputs [40, 57]. As a consequence, the description of TMS findings observed in the different VCI subtypes cannot be linked to behavioral changes. It is worth to highlight that the available TMS evidences in vascular-related cognitive disorders point out the possibility to identify a profile of cortical excitability related to each VCI subtype rather than a specific behavioral deficit. On the other hand, behavioral changes are common in all VCI patients, and therefore, the identification of a distinctive TMS correlate is challenging. On the contrary, studies in vascular depression allowed us to identify a more specific pattern of cortical excitability, thus suggesting the role of some TMS measures as putative markers of disease process and progress compared to nondepressed subjects.
- The available results on relatively small sample size might not be confirmed on larger populations, although most of them have been obtained from homogeneous samples in terms of demographics, clinical and neuroradiological features, and age matching with healthy controls.
- The hypothesis to identify a characteristic signature in patients with subcortical vascular disease at risk for developing VaD or mixed dementia could be risky given the paucity of previous data and the difficulty of similar approaches in other dementing conditions, such as non-AD dementias; consequently, the identification of a pattern of cortical excitability rather than single marker of the disease process and progress would be more reliable and reproducible [34].
- As known, vascular lesions, even in the absence of any motor deficit, give a significant contribution to the development and progression of degenerative dementia, so that it cannot be excluded that some patients enrolled in the studies here reviewed had a mixed form of dementia rather than a pure VaD. In this respect, TMS is not currently able to clearly distinguish VaD from AD based only on their neurophysiological profile or to clearly disentangle the vascular from degenerative burden [30].
- Elderly population usually takes a number of drugs for the treatment/control of vascular risk factors (i.e., antithrombosis agents, antihypertensive drugs, statins, and oral antidiabetes therapy) which may affect the measures of cortical excitability and conductivity. The same holds for psychotropic drugs often taken for the treatment of anxiety and depressive disorders (i.e., benzodiazepines, antidepressants, antipsychotics, and mood stabilizers) [44, 45, 57]. Therefore, the study design should consider patients under medicaments which, to the best of current knowledge, have no or limited influence at the level of TMS.
- Finally, a usual limitation in TMS research is the threshold of sensitivity and specificity of the TMS measures when used as a diagnostic tool. In order to adequately define sensitivity and specificity, the individual measures in all the patients and controls, not only the mean values, would be required. Moreover, to estimate the number of false positives, the whole subject population needs to be followed up independently to assess if they show clinical deterioration [34]. Up to now, very few studies fulfill these requirements, and the application of TMS in dementia needs future studies with methodological improvement and high degree of standardization. Nevertheless, in view of the exponential growth of new and sometimes not concordant data, this review aimed to consolidate, wherever possible, the available knowledge on TMS and VCI.

## 6. Conclusion, Translational Value, and Future Perspectives

To date, the development of dementia cannot be accurately predicted by conventional investigations [115]. However, unlike degenerative dementias, VaD can be prevented, at least in part, in most of the patients through a careful prevention and a close monitoring of vascular risk factors. Preventing VCI means to prevent vascular accidents and future dementia, to reduce mortality, disability, and institutionalization rates, to maintain an acceptable functional status in the elderly, and, ultimately, to save clinical-social costs [116].

The effect of conventional treatment on VCI and VaD is not well established. Moreover, the heterogeneous construct of VCI constitutes a challenge in the selection of appropriate outcome measures in clinical trials of pharmacological interventions. Therefore, the discovery of new early targets to prevent or treat VaD is desirable. In this context, despite the fact that patient cohorts and methodologies are not always homogeneous between studies, much of the literature agrees on the utility of TMS in dementias [34]. Although a single measure is not sufficient to define a diagnosis, all together the parameters of interest are footprints of specific pathophysiological processes that affect motor and nonmotor areas in various forms of dementia. Taken together, the available TMS evidences point out the possibility to identify a specific profile of cortical excitability each related to a VCI subtype, and in particular to predict conversion of the so-called “brain at risk” for VaD into an overt dementia. This will be of pivotal importance when designing trials of disease-modifying drugs and innovative nonpharmacological approaches based on brain stimulation. Conversely, in patients with overt dementia, TMS can be exploited to select and evaluate the responders to specific drugs and might also become a rehabilitative tool in the attempt to restore impaired brain plasticity.

Although it is not possible at the moment to determine whether findings from the studies here reviewed are reflected in decision-making in the care of patients, TMS might prompt a design of an instrument for screening of population at risk, studying drug-induced changes in the electrical properties of the human cortex, probing models of brain connectivity, and testing neuromodulatory therapeutic tools for cognitive rehabilitation. When patients at risk are identified, a more careful prevention and control of vascular risk factors should be considered. Further longitudinal studies combining TMS with other neurophysiological techniques (including high-density EEG and event-related potentials) and advanced structural and functional imaging data (such as functional MRI and diffusion tensor imaging), as well as serum and CSF biological markers will clarify the impact of vascular cognitive, mood, and behavioral deficits on cortical excitability and synaptic plasticity.

## Figures and Tables

**Table 1:** TMS studies in patients with vascular cognitive impairment.

Study	Participants (mean age ± SD, years)—M/F	VCI subtype	Diagnostic criteria	MRI correlates	CNS active drugs	TMS technique/type of coil/muscle(s)	Main TMS findings	Translational value
Alagona et al. [80]	20 VCI patients (71.80 ± 9.37)—8/12 20 AD patients (72.20 ± 7.53)—7/13 20 controls (68.55 ± 7.96)—8/12	Subcortical ischemic VaD	NINDS-AIREN NINDS-ADRDA	Subcortical lacunes and/or multiple involvement of the inner white matter	NR	Single-pulse TMS/circular coil/first dorsal interosseus	↓ rMT in VCI (++) and AD (+) = MEP/CMAP = CSP	Increased cortical excitability in both subcortical ischemic VaD and AD compared to healthy controls (especially VaD versus controls).

Di Lazzaro et al. [81]	12 VCI patients (70.9 ± 9.6)—8/4 12 AD patients (69.3 ± 7.3)—NR 12 controls (73.1 ± 5.4)—NR	Subcortical ischemic VaD	NINDS-AIREN NINDS-ADRDA	Small-vessel disease with multiple lacunar infarcts (<2 cm in size)	No	Single-pulse TMS + SAI protocol/ figure-of-eight coil/first dorsal interosseous	= rMT, aMT = SAI in VaD ↓ SAI in AD (abnormal SAI in 75% of AD and in 25% of VaD patients)	Normal central cholinergic circuit functioning in most of the patients with subcortical ischemic VaD in contrast with those with AD.

Nardone et al. [82]	20 patients (70.9 ± 4.4)—12/8 25 controls (71.8 ± 5.6)—14/11	Subcortical ischemic VaD	NINDS-AIREN	9 with predominant lacunar infarctions, 11 with predominant WMLs	No	Single-pulse and paired-pulse TMS + SAI protocol/ figure-of-eight coil/first dorsal interosseous	↓ SAI in VaD = rMT, aMT = CMCT = CSP = SICI, ICF	Mean cholinergic activity significantly reduced in patients, although individual data varied widely (from normal to markedly reduced).

Manganelli et al. [83]	10 patients (70.9 ± 4.4)—5/5 10 controls (71.8 ± 5.6)—5/5	CADASIL	Mutation in the exon 19 *Notch3* gene	Bilateral WMLs with involvement of the external capsule	NR	Single-pulse TMS + SAI protocol/ figure-of-eight coil/first dorsal interosseous	↓ rMT ↓ SAI	Impairment of cholinergic activity in patients compared to controls.

Pennisi et al. [84]	20 VaD patients (71.8 ± 9.4)—8/12 20 VCI-ND patients (65.9 ± 10.6)—8/12 20 controls (68.5 ± 7.9)—8/12	Subcortical ischemic VaD VCI-ND	NINDS-AIREN	Lacunar state and/or WMLs	No	Single-pulse TMS/circular coil/first dorsal interosseus	↓ rMT in VaD (++) and VCI-ND (+) ↑ MEP amplitude in VaD	Enhanced motor cortex excitability only in patients with dementia and not in those without dementia and controls.

Nardone et al. [85]	28 patients (69.5, range 57–79)—17/11 20 controls (69.2, range 55–79)—13/7	Subcortical ischemic VaD	Research criteria for subcortical VaD	Cerebral microbleeds, WMLs, lacunar infarction	No	Single-pulse and paired-pulse TMS + SAI protocol/ figure-of-eight coil/first dorsal interosseous	= rMT = CMCT = SICI, ICF ↓ SAI in patients with microbleeds =rMT after donepezil ↑ SAI after donepezil	The impact of microbleeds on central cholinergic function was independent of the extent of associated white matter changes and ischemic stroke.

Bella et al. [86]	15 VCI-depressed patients (70.5 ± 6.6)—7/8 10 VCI-nondepressed patients (70.8 ± 6.3)—6/4 10 controls (67.7 ± 3.9)—5/5	VCI-ND	DSM-IV-TR for dementia and MDD Research criteria for subcortical VaD	Subcortical vascular disease with predominant WMLs	No	Single-pulse and paired-pulse TMS/figure-of-eight coil/first dorsal interosseous	= rMT = CSP = CMCT = SICI ↑ ICF	The neurophysiological mechanisms underlying vascular depression differ from those reported in major depressive disorder and seem to be similar to those of the patients with subcortical ischemic vascular disease.

Bella et al. [87]	10 patients (70.8 ± 6.32)—6/4 10 controls (67.7 ± 3.92)—5/5	VCI-ND	DSM-IV-TR for dementia Research criteria for subcortical VaD	Subcortical vascular disease with predominant WMLs	No	Single-pulse and paired-pulse TMS/ figure-of-eight coil/first dorsal interosseous	= rMT = CSP = CMCT = SICI, ICF	Evidence of functional changes in intracortical excitatory neuronal circuits in patients compared to controls.

Bella et al. [88]	9 patients (median 70, range 66–84)—NR 9 controls (median 67, range 65–88)—NR	VCI-ND	DSM-IV-TR for dementia Research criteria for subcortical VaD	Subcortical vascular disease with predominant WMLs	No	Single-pulse and paired-pulse TMS/figure-of-eight coil/first dorsal interosseous	↓ rMT at follow-up No significant difference for the other electrophysiological measures at follow-up	Increased cortical excitability in patients during the progression of cognitive impairment, suggesting plasticity as a compensatory mechanism.

Lanza et al. [89]	15 patients (71.40 ± 5.53)—9/6 15 controls (68.67 ± 3.62)—9/6	VCI-ND	DSM-IV-TR for dementia Research criteria for subcortical VaD	Subcortical vascular disease with predominant WMLs	No	Single-pulse TMS/figure-of-eight coil/first dorsal interosseous	= rMT = CSP, iSP = MEP = CMCT	Unlike mild cognitive impairment and degenerative dementia, transcallosal inhibitory functioning was preserved in VCI-ND.

List et al. [90]	20 patients (72, range 63–78)—9/11 20 controls (71, range 60–77)—10/10	VCI-ND	Research criteria for subcortical VaD	Severe ischemic subcortical vascular disease	No	Single-pulse TMS + PAS protocol/figure-of-eight coil/abductor digiti minimi	= rMT = MEP at baseline ↑ MEP amplitude to PAS in 80% of controls and 50% of patients	Enhanced cortical plasticity as compensatory mechanism to counteract memory decline in severe subcortical small vessel disease.

Palomar et al. [91]	10 patients (56.9 ± 9.8, range 36–70)—5/5 10 controls (57.0 ± 10.3, range 39–71)—4/6	CADASIL	Mutation in the exon 19 *Notch3* gene	Leukoaraiosis, external capsule and anterior temporal pole T2-hyperintensities, lacunar infarcts	No	Single-pulse and paired-pulse TMS + SAI and PAS protocols/figure-of-eight coil/first dorsal interosseous and abductor pollicis brevis	= rMT = CSP = SICI, LICI ↓ ICF ↓ SAI ↓ MEP facilitation after PAS	Acetylcholine and glutamate might be involved in CADASIL; abnormal sensory-motor plasticity correlated with the neuropsychological profile.

Concerto et al. [76]	11 VCI-depressed patients (57.72 ± 3.20)—6/5 11 MDD patients (57.18 ± 7.10)—5/6 11 controls (67.36 ± 3.70)—6/5	VCI-ND	DSM-IV-TR for dementia and MDD Research criteria for subcortical VaD	Periventricular and deep WMLs or multiple lacunes in deep grey matter, and at least moderate WMLs	None in VCI group; all in MDD group	Single-pulse and paired-pulse TMS/figure-of-eight coil/first dorsal interosseous	↑ rMT (>left hemisphere) in MDD ↑ CSP (>left hemisphere) in MDD = MEP = CMCT = SICI, ICF	Distinctive patterns of motor cortex excitability between late-onset depression with subcortical vascular disease and early-onset recurrent MDD without vascular lesions.

Nardone et al. [92]	8 VCI patients (72.5 ± 6.1)—NR 8 AD patients (72.5 ± 6.1)—NR 8 controls (72.5 ± 6.1)—NR	CADASIL	Mutation in the exon 19 *Notch3* gene NINDS-ADRDA	NR	No	Single-pulse and paired-pulse TMS + SAI protocol/figure-of -eight coil/first dorsal interosseous	= rMT = SICI, ICF ↓ SAI in all patients ↑ SAI after L-Dopa in AD patients only	Significantly reduced cholinergic circuit functioning in all patients, with restoration by L-Dopa in AD subjects only.

List et al. [93]	12 patients (62.0 ± 14.0, range 30–74)—9/3 10 controls (66.8 ± 5.9)—NR	3 patients with poststroke VCI-ND	ECST criteria for unilateral internal carotid artery high-grade stenosis or occlusion	Left anterior cerebral artery infarction; right internal capsule infarction; left anterior cerebral artery infarction; NR in the other patients	No	Single-pulse and paired-pulse TMS + PAS protocol/figure-of-eight coil/abductor pollicis brevis	In the affected hemisphere: ↑ rMT ↓ CSP ↓ LTP-like plasticity = motor learning between hemispheres	Despite decreased LTP-like plasticity in the affected hemisphere, motor learning was comparable between hemispheres, possibly due to GABA-B-mediated cortical motor excitability changes within the affected side.

Guerra et al. [94]	7 VCI patients (78.1 ± 4.3)—2/5 9 AD patients (74.3 ± 7.7)—0/9 9 controls (75.0 ± 15.5)—3/6	Subcortical ischemic VaD	NINDS-AIREN NINDS-ADRDA	High degree of white matter hyperintensities	No	Single-pulse TMS + mapping study/figure-of-eight/extensor digitorum communis and abductor digiti minimi	↓ rMT in AD (++) and VaD (+) ↑ area and volume of muscular areas in AD (++) and VaD (+) Medial-frontal shift of the center of gravity map in all patients.	Similarly enhanced cortical excitability and plasticity in AD and VaD; the hyperexcitability can promote cortical plasticity, likely playing a compensatory function.

Bella et al. [95]	25 patients (67.50 ± 6.72)—10/15 20 controls (64.60 ± 7.67)—9/11	VCI-ND	DSM-IV-TR for dementia Research criteria for subcortical VaD	Subcortical vascular disease with predominant WMLs	No	Single-pulse TMS + SAI protocol/figure-of-eight coil/first dorsal interosseous muscle	= rMT = CSP = MEP = CMCT ↓ SAI, but not after Bonferroni correction	Unlike degenerative dementia, central cholinergic pathway was not clearly involved in patients with leukoaraiosis compared to controls.

Pennisi et al. [96]	16 depressed patients (68.1 ± 8.6)—NR 11 VCI-nondepressed patients (70.0 ± 7.0)—NR 15 controls (63.8 ± 7.2)—NR	VCI-ND	DSM-IV-TR for dementia and MDD Research criteria for subcortical VaD	Subcortical vascular disease with predominant WMLs	No	Single-pulse and paired-pulse TMS/figure-of-eight coil/first dorsal interosseous	↑ ICF in nondepressed at baseline ↓ rMT in all patients at follow-up ↑ CSP only in controls at follow-up = ICF in all subjects at follow-up	The mechanisms enhancing the risk of dementia in patients with vascular depression might be related either to subcortical vascular lesions or to the lack of compensatory functional cortical changes.

AD = Alzheimer's disease; aMT = active motor threshold; CADASIL = cerebral autosomal dominant arteriopathy with subcortical infarcts and leukoencephalopathy; CMAP = compound motor action potential; CMCT = central motor conduction time; CSP = contralateral cortical silent period; DSM-IV-TR = Diagnostic and Statistical Manual of Mental Disorders, Fourth Edition, Text Revision; ECST = European Carotid Surgery Trial; F = females; GABA = gamma-aminobutyric acid; ICF = intracortical facilitation; iSP = ipsilateral silent period; L-Dopa = L-3,4-dihydroxyphenylalanine; LICI = long-latency intracortical inhibition; LTP = long-term potentiation; M = males; MDD = major depressive disorder; MEP = motor-evoked potential; MMSE = Mini Mental State Examination; MDD = major depressive disorder; MRI = magnetic resonance imaging; NINDS-ADRDA = National Institute of Neurological and Communicative Disorders and Stroke and the Alzheimer's Disease and Related Disorders Association; NINDS-AIREN = National Institute for Neurological Disorders and Stroke-Association Internationale pour la Recherche et l'Enseignement en Neuroscience; NR = not reported; PAS = paired-associative stimulation; rMT = resting motor threshold; SAI = short-latency afferent inhibition; SD = standard deviation; SICI = short-latency intracortical inhibition; TMS = transcranial magnetic stimulation; VaD = vascular dementia; VCI = vascular cognitive impairment; VCI-ND = vascular cognitive impairment-no dementia; WMLs = white matter lesions; ↑ = increase; ↓ = decrease; = no significant difference. Research criteria for subcortical VaD [97].
